# Adhesion evaluation of a new self-cured resin to sclerotic dentin

**DOI:** 10.1590/0103-644020256485

**Published:** 2025-09-19

**Authors:** Paulo Henrique dos Santos Belo, Thiago Espindola Morais da Silva, Wendell de Sousa Loterio, Gustavo Miranda Rocha, Cesar dos Reis Perez

**Affiliations:** 1 Prosthesis Department, School of Dentistry of the State of Rio de Janeiro(UERJ), Rio de Janeiro, RJ, Brazil.; 2 Unidade de Pesquisa Urogenital, Anatomy Department - Medical Sciences Faculty of the State of Rio de Janeiro(UERJ), Rio de Janeiro, RJ, Brazil

**Keywords:** Dentin-bonding agents, dental adhesives, composite resins, sclerotic dentin, dental air abrasion

## Abstract

Recently, a self-curing dental composite (Stela) was developed, promising several advantages and indications. One of them includes the restoration of Class V cavities. However, non-carious cervical lesions (NCCLs) frequently present sclerotic dentin as a substrate for adhesion. Thus, the objective of this *in vitro* study was to evaluate the bond strength of this material on bovine sclerotic dentin through a shear bond test (SBS), comparing it with a universal self-etching adhesive system with 10-MDP, which is considered to have the best adhesive performance in this situation (Single Bond Universal). In parallel, the effect of airborne-particle abrasion (APA) on the performance of the two adhesive systems was evaluated and whether it affected them differently. Four groups were tested: Stela (S), APA + Stela (APAS), Universal (U), and APA + Universal (APAU), each one with 12 specimens. In addition, the type of fracture and scanning electron microscopy (SEM) of representative specimens of the groups were evaluated. Shapiro-Wilk indicated a normal distribution of the data. The results were evaluated using two-way ANOVA, with a significance level of 5%. The analysis showed no statistically significant difference in the performance of the two adhesives. Additionally, APA increased the adhesive strength of both adhesives, and there was no significant interaction between APA and the two adhesives tested. The results obtained by analyzing the fracture type and SEM also showed a similar pattern. Stela Primer showed SBS values to sclerotic dentin similar to Single Bond Universal.

## Resumo

Recentemente, foi desenvolvido um compósito odontológico auto polimerizável (Stela), prometendo diversas vantagens e indicações. Uma delas inclui a restauração de cavidades tipo Classe V. Entretanto, lesões cervicais não cariosas (LCNCs) frequentemente apresentam dentina esclerótica como substrato para adesão. Assim, o objetivo deste estudo *in vitro* foi avaliar a resistência de união deste material à dentina esclerótica bovina por meio de um teste de cisalhamento, comparando-o com um sistema adesivo universal auto condicionante com 10-MDP, considerado de melhor desempenho adesivo nesta situação (Single Bond Universal). Paralelamente, foi avaliado o efeito da abrasão por jateamento de partículas (APA) sobre o desempenho dos dois sistemas adesivos e se ela os afetou de forma diferente. Quatro grupos foram testados: Stela (S), APA + Stela (APAS), Universal (U) e APA + Universal (APAU), cada um com 12 espécimes. Além disso, foram avaliados o tipo de fratura de todos os espécimes e a microscopia eletrônica de varredura (MEV) de espécimes representativos dos grupos. O teste Shapiro-Wilk indicou uma distribuição normal dos dados. Os resultados foram avaliados por Two-way ANOVA, com nível de significância de 5%. A análise não mostrou diferença estatisticamente significativa entre o desempenho dos dois adesivos, que o APA aumentou a força adesiva de ambos os adesivos e que não houve interação diferente entre o APA e os dois adesivos testados. Os resultados qualitativos obtidos pela análise do tipo de fratura e MEV também mostraram um padrão semelhante. Stela Primer mostrou resultados de SBS sobre dentina esclerótica similares ao Single Bond Universal.

**Figure:**
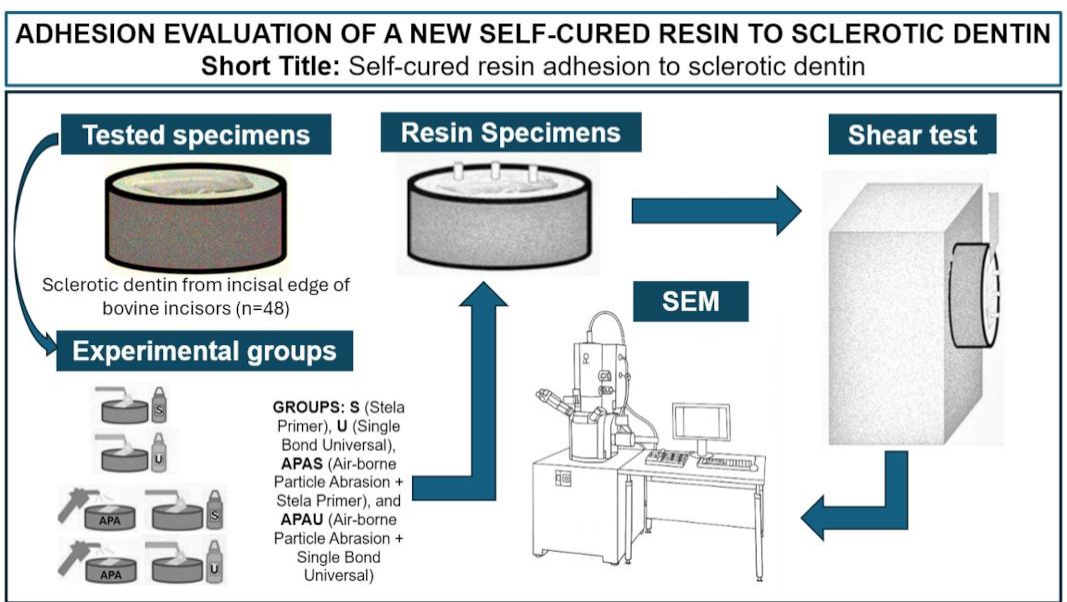

## Introduction

Recently, a self-curing, bulk-fill restorative material has been introduced in the market (Stela AUTOMIX, SDI, Australia), comprising an adhesive system that requires no light curing, as it polymerizes upon contact with the restorative material. The primer contains a catalyst that initiates the curing process at the restoration interface. Thus, it would provide a gap-free interface and reduce contraction-related problems. Also, it is believed to have a polymerization sequence that mitigates stress. Additionally, it presents a chameleon effect that mimics the shade of the surrounding teeth. It is indicated for various clinical applications, including the restoration of Class V cavities ^1^ ^,^ ^2^. This type of cavity presents a sclerosed dentinal substrate, which can interfere with the performance of the adhesive system and, consequently, generate critical problems at the tooth-restoration interface ^3^. A recent in vitro study demonstrated the good performance of Stela Primer in restorations of occlusal Class I cavities in freshly cut and healthy dentin ^2^.

Considering that the adhesive system used for Stela resin is specific (Stela Primer/SDI) and incompatible with other types of resin, it is relevant to compare the adhesive performance of this system with that considered to have presented the best performance on this type of substrate both in vitro ^4^ and clinically ^5^ ^,^ ^6^ ^,^ ^7^ ^,^ ^8^, in this case, the universal self-etching adhesives with 10-MDP in the composition ^9^.

Another aspect that still generates controversy-the roughening of the sclerotic dentin prior to adhesive application-also warrants testing to understand better its effect on the performance of this specific adhesive system ^10^ ^,^ ^12^ ^,^ ^13^. On the one hand, in vitro studies ^4^ ^,^ ^10^, a systematic literature review ^11^, and a systematic review ^13^ argue that roughening the surface of sclerotic dentin and even healthy dentin increases adhesion to these substrates. On the other hand, other studies, such as a recent systematic review ^12^, consider the results inconclusive. The studies revealed high heterogeneity, indicating that additional clinical trials are necessary to determine the optimal dentin treatment option for NCCLs.

Therefore, this study aimed to evaluate the *in vitro* adhesive performance of this new self-cured adhesive (Stela Primer) over sclerotic dentin, with or without previous air-particle abrasion (APA), through a shear bond strength test, and scanning electron microscopy (SEM), comparing the results with a universal self-etch adhesive with 10-MDP (Single Bond Universal/3M). Three null hypotheses were formulated: that there will be no significant difference in the shear bond strength of the two adhesive systems, that the previous airborne particle (APA) will not influence the shear bond strength of the two adhesive systems, and that the APA did not influence the two adhesives tested differently.

## Material and Methods

### Ethical Considerations

The Ethics in Research Committee of the Pedro Ernesto University Hospital/UERJ approved the research project (CAAE:** **10522918.2.0000.5259).

### Sample Calculations and Acquisition

After a pilot study with five specimens per group, the following parameters were considered for the sample calculation: the minimum difference between treatment means = 0.38, standard deviation of the error = 0.5, number of treatments = 4, test power = 0.80, and significance level = 0.05. Thus, 48 bovine central incisors were used (n = 12).

The teeth were obtained from a slaughterhouse, and specimens with natural dentin exposure caused by advanced incisal border edge wear were chosen. After extraction, all teeth were cleaned from adhered soft tissues using #15 surgical blades (Swann-Morton, Sheffield, England) and stored in distilled water at 4^o^ C until one week before being used in the experiment. The roots were sectioned, and the coronal pulp was removed. The teeth were examined with 10X magnification, and specimens presenting fractures or cracks were excluded and replaced by others. The bovine substrate was used instead of human teeth, as it is readily available and has been considered a reliable substitute ^14^. The use of human teeth in laboratory research is restricted due to ethical limitations, difficulty obtaining the appropriate sample size, and the impossibility of standardization. For these reasons, the necessity for tooth substrates to replace human teeth for in vitro studies is increasing. Bovine dentine is used as a substitute for human dentine in adhesion tests because it has a collagen organic matrix similar to humans ^15^.

### Sample Preparation

The teeth were then embedded in a chemically activated resin poured into a sectioned PVC pipe so that the dentin surface was exposed to the external surface of the test specimen, facing the surface of the acrylic resin. The specimens were randomly assigned to the four experimental groups (n = 12) through group drawing. They were flattened and wet polished with 200, 320, 400, and 600-grit SiC paper (Norton S.A., Sao Paulo, SP, Brazil) for 30 seconds each disc. All the specimens received prophylaxis with pumice and then were ultrasonicated for 30 seconds. After these procedures, the specimens were analyzed under a stereoscopic magnifying glass with 10X magnification regarding the appearance of the superficial dentin to observe whether they presented an appearance compatible with Category IV in the classification of Heymann & Bayne (1993). In this, significant sclerosis is present. Dentin is dark yellow or even discolored (brownish) and appears glassy, with significant translucency or transparency evident ^16^.

From this point on, the specimens were randomly distributed among the groups representing different types of adhesive treatment: Stela Primer (S), airborne-particle abrasion (APA) plus Stela Primer (APAS), Single bond Universal (U), and roughening through sandblasting plus Single bond Universal (APAU). The materials used are listed in Box 1. The adhesive protocols are detailed in Box 2.

**Box 1:**
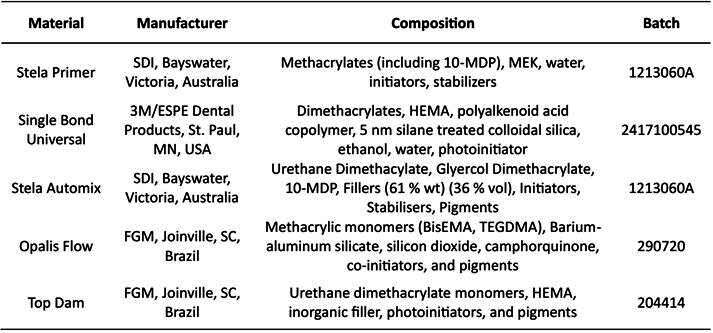
Materials, manufacturer, composition, and batch number.

Each dentinal surface received two or three cylinders of flowable resin (Opalis Flow; FGM) or Stela (SDI). After each specific treatment, the resin cylinders were made directly on the sclerotic dentin surface. The preparation of the specimens for the shear bond strength test (SBS) was carried out using cylindrical matrices with an internal diameter of 2 mm and a height of 2 mm, obtained from the sectioning of triple Oblate-type hoses for dental equipment. The cylindrical matrices were fixed perpendicularly to the dentin surface with a gingival barrier (Top Dam, FGM) and polymerized for 30 seconds with a dental polymerization light (Radii Cal, SDI) with an intensity of 1200 mW/cm^2^. Each matrix was filled with a compatible flowable resin (Opalis/FGM, which was used for the U and APAU groups, and Stela/SDI, which was used for the S and APAS groups). All the excess resin covering the walls of the matrices was removed carefully to allow a predictable removal after curing. Opalis specimens were light-cured for 40 seconds, with the emission light directly supported by the cylindrical matrices. Stela specimens were covered with a thin layer of glycerin to prevent the formation of an oxygen-inhibition superficial layer. This oxygen inhibition is characteristic of the material. Still, it does not interfere with its clinical use, as in most clinical situations, it is recommended to clean the inhibitory layer and finish with burs and a water spray, followed by polishing. In the case of making the test specimens, glycerin gel was used since the surface layer was not removed.

**Box 2:**
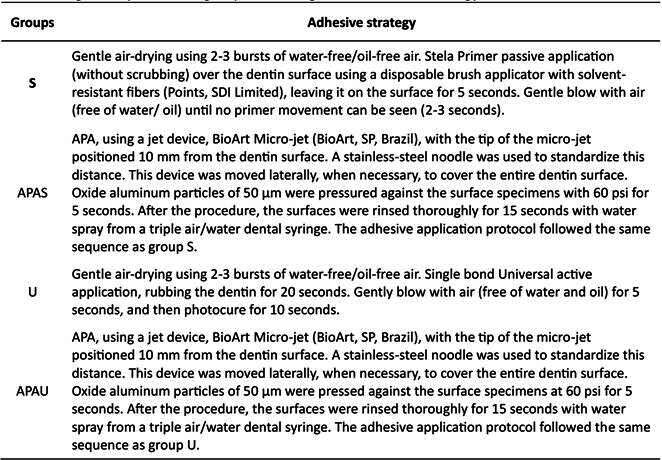
Design of experimental groups according to the adhesive strategy.

The specimens were stored in distilled water for 24 hours. A scalpel, blade no. 15, was used to remove the gingival barrier resin, and the matrices were gently removed by pulling upwards. The matrix was cut with a scalpel blade if any resistance was observed. The integrity and quality of the cylinder were evaluated for the absence of bubbles, voids, or excess flowable resin beyond the cylinder and the presence of a 90-degree angle between the tooth surface and the resin cylinder. After testing, a digital micrometer (Absolute CD-6 CSX-B; Mitutoyo Sul American, Suzano, SP, Brazil) was used to measure the cylinder diameters. Any specimen with dimensional discrepancies was discarded. Eventually, the resin cylinders constructed from the insertion into the silicone cylindrical matrices would come loose during its removal or sectioning (when necessary). In all cases in which this occurred (eight cylinders in total), it was possible to observe that there was a failure in the insertion of the flowable resin since bubbles were present in the region of the adhesive interface, probably due to the intrinsic difficulty of insertion and the occlusion of air. Despite this, each surface represented a specimen, and the final resistance obtained by each specimen in the shear test resulted from the average of two or three cylinders fixed to each surface. There was no case in which only one cylinder remained after the removal of the silicone matrix.

### Shear bond strength test and failure analysis

**Figure 1:**
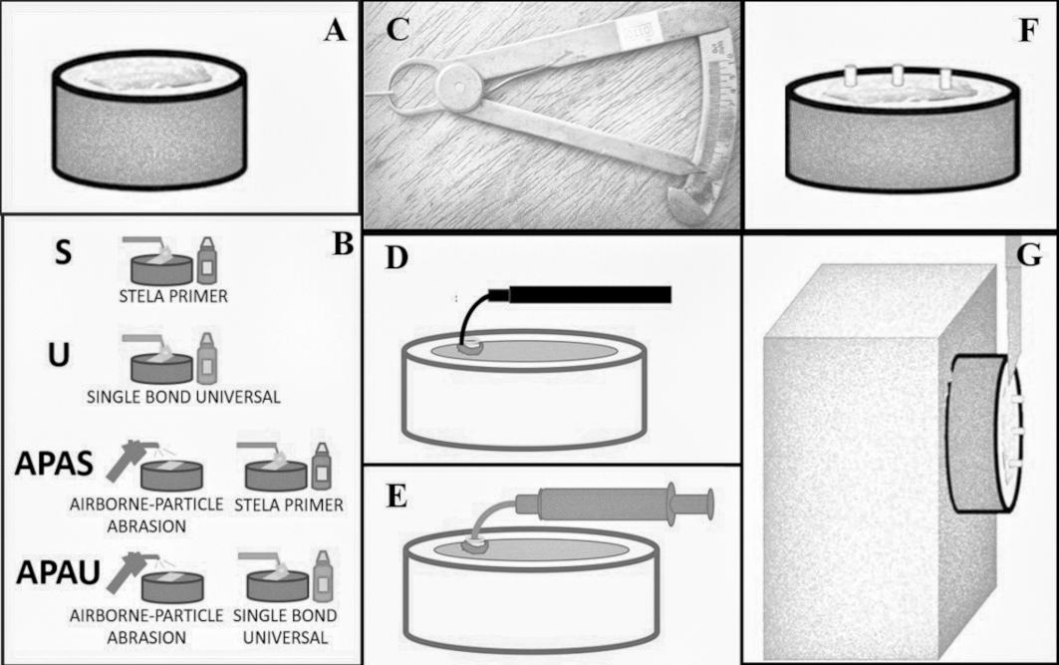
Schematic drawing shows specimen preparation for SBS. (A) The roots of all bovine teeth were removed by sectioning at the cementum-enamel junction. The crowns were embedded in PVC tubes filled with acrylic resin so that the incisal surface was exposed at the same level as the acrylic resin. This surface was ground from coarser to finer on wet # 180, 400, 600, and 1000 grit sandpapers (60 seconds), washed with water, and gently blotted dry with absorbent paper; (B) schematic representation of used protocols; (C) measuring the diameter of the active tip of the Stela auto mix system; (D) gingival barrier application; (E) insertion of the flowable resin; (F) resin specimens ready for testing after matrices removal; (G) specimens positioned for the SBS test.

Each PVC tube with a bovine tooth was placed upright on a support base, allowing the cylinders to be unsupported, and the adhesive interface was positioned perpendicular to the shearing force. A shear test probe was attached to a universal testing machine (EMIC DL2000; Instron Brazil) with a 50 N load cell and tested under shear at 0.5 mm/min until failure. The test probe was designed so that its active edge presented a concave notch compatible with the diameter of the cylinder to be tested to induce a more uniform transmission of shear force throughout its cross-section ^17^ ^,^ ^18^ ^,^ ^19^ **.** Figure 1 shows a schematic representation of the specimens' preparation. The arithmetic means of the sum of all the results obtained on each cylinder was computed for each tooth surface (n = 12).

The specimens' failure modes were classified as adhesive, dentin cohesive, resin cohesive, mixed, or premature failure (if the specimen was lost before the test). The classification was done under 10x magnification using a stereomicroscope (Olympus SZ40, Tokyo, Japan).

### Scanning electron microscopy (SEM)

For SEM, representative samples of each group (n = 2) were prepared according to the adhesive protocol for each group, as described earlier. For this evaluation, however, cubic resin blocks were created directly on the dentin after each adhesive procedure. Then, the specimens were sectioned with a diamond disc in an Isomet 1000 saw (Buehler, Dusseldorf, Germany), with a speed of 150-200 rpm under continuous water cooling to obtain a flat sectioned area involving the composite resin, the sclerotic dentin, and the adhesive interface, and, then ultrasonic cleaned in distilled water for 3 min to remove debris.

The specimens were covered with gold in a vacuum-metallizing machine (SCD 050, Bal-Tec AG, Balzers, Liechtenstein) at a pressure of 0.01 mbar, current of 40 mA, working distance of 50 mm, coating time of 90 seconds, and mean coating thickness of 20 to 30 nm. Images were taken via low-vacuum scanning electron microscopy (SEM) using the electron backscattered technique (15kV, TM3030Plus Tabletop Microscope, Hitachi, Tokyo, Japan).

**Figure 2:**
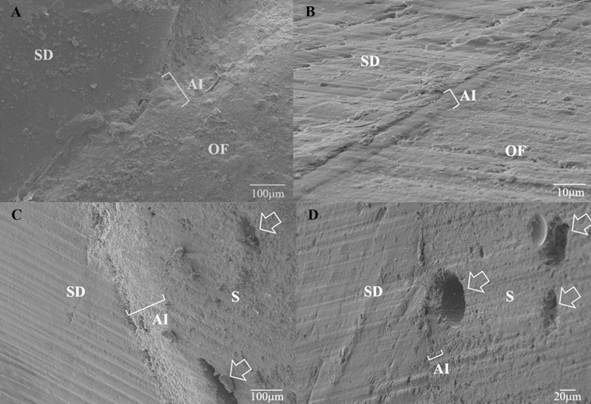
Representative photomicrographs of the adhesive interface of each group tested: U (A), APAU (B), S (C), and APAS (D). The respective acronyms represent AI (adhesive interface), HL (hybrid layer), OF (Opalis flow), and S (Stela). The arrows show bubbles within the Stela resin. Scale bars are presented in the lower right corner of each photomicrograph.

### Statistical Analysis

The data collected were statistically analyzed using the SPSS software (version 22) for Windows (SPSS Inc., Chicago, IL, USA). Shapiro-Wilk indicated a normal distribution of the data. Therefore, the bond strength values were compared using Two-way ANOVA. A significant level of 5% was established.

## Results

Table 1 presents the bond strength values, mean, and standard deviation (expressed in MPa) according to the groups. Table 2 shows the test between subjects obtained after Two-way ANOVA. The "adhesive" factor did not significantly influence the results (P = 0,22), demonstrating that the adhesives performed similarly. On the other hand, the factor "APA" presented significant differences (P = 0,01), demonstrating the effectiveness of airborne-particle abrasion in increasing adhesive shear bond strength values. The interaction between the two factors (adhesive and APA) did not significantly influence the results (P = 0,48), demonstrating that the blasting did not influence the two adhesives tested differently.

Figure 2 shows selected micrographs of specimens of the four groups. Qualitative analysis of the images revealed similar adhesive interfaces. A relevant observation was the occurrence of bubbles in the Stela resin specimens.

Table 3 shows the percentage (%) of failure types for each experimental group. Most failures were adhesive in all groups evaluated. Mixed failures, with small portions of resin detached after fracture, were observed in all groups.

**Table 1:** Shear bond strength values (MPa) for experimental groups (mean ± standard deviation).

Adhesive	APA	Mean (SD)	N
Stela	no (S)	13,01±4,66	12
	yes (APAS)	19,21±9,36	12
>Single Bond Universal	no (U)	12,06±3,17	12
	yes (APAU)	15,62±6,53	12

**Table 2:** Test of between-subjects effects considering the dependent variable “shear bond strength”

Source	F	Sig.
Adhesive	1,53	,22
APA	7,06	,01
Adhesive*APA	,51	,48

**Table 3:** percentage (%) of failure types for each experimental group.

Group	Failure type	%
S	adhesive	84
	cohesive dentin	0
	cohesive resin	0
	mixed	16
>APAS	adhesive	68
	cohesive dentin	0
	cohesive resin	0
	mixed	32
>U	adhesive	82
	cohesive dentin	0
	cohesive resin	0
	mixed	18
>APAU	adhesive	64
	cohesive dentin	0
	cohesive resin	0
	mixed	36

S: Stela Primer, APAS: airborne-particle abrasion before Stela Primer, U: Single Bond Universal, APAU: airborne-particle abrasion before Single Bond Universal

## Discussion

This study evaluated the shear bond strength between two adhesive systems (Stela/SDI and Single Bond Universal/3M) to sclerotic dentin, with or without previous airborne particle abrasion. Considering the three null hypotheses formulated, the first was accepted, as there were no statistically significant differences between the adhesive systems; the second was rejected as the APA induced statistically significant superior SBS results in both adhesive systems; and the third was accepted as the APA did not influence the two adhesives tested differently.

The use of a self-etching adhesive with 10-MDP in its composition as a comparator follows the current trend that considers this to be the adhesive type that presents the best performance in sclerotic dentin and the restoration of Class V cavities or non-carious cervical lesions ^5^ ^,^ ^6^ ^,^ ^7^ ^,^ ^8^ ^,^ ^9^. In the present study, the chemically activated adhesive system recently introduced to the market, Stela Primer, performed similarly to Single Bond Universal, with or without prior application of primer activator (APA). Since the self-cure bulk-fill restorative material (Stela/SDI) is a recent launch and few studies are available, and since the manufacturers indicate its use for restoring Class V cavities and NCCLs, this study sought to study the behavior of its adhesive system on sclerotic dentin, a common type of substrate for adhesion in these types of clinical situations. The results confirm the good performance proposed by the manufacturers. They can be considered justifiable since their specific adhesive system (Stela Primer/SDI) also contains the adhesive monomer 10-MDP in its composition.

The incorporation of APA as an additional variable is due to the lack of consensus regarding the favorable cost-benefit ratio of its use. In this study, the previous use of APA significantly affected the shear bond strength to sclerotic resin, improving the resistance values for both adhesives tested. This performance is consistent with other articles, mainly in vitro studies ^10^ ^,^ ^11^ ^,^ ^12^ ^,^ ^13^. However, better adhesive performance *in vitro* studies did not necessarily represent better results in clinical studies. Although most of these studies had short follow-up periods, the concurrence of multiple variables found clinically makes it challenging to obtain practical conclusions ^20^ ^,^ ^21^. Thus, some articles tend to present a more conservative approach, indicating new studies with extended follow-up periods to gather consistent evidence ^22^ ^,^ ^23^ ^,^ ^24^.

The fractographic analysis of the type of fracture obtained relatively homogeneous results for both adhesive systems. In the groups with previous APA, there was a tendency for an increase in the occurrence of mixed failures, which is compatible with the increase in adhesive strength observed in these groups.

Similar adhesive interfaces were observed independently with or without previous APA in the SEM evaluation. A relevant observation was the occurrence of microbubbles (or voids) in the Stela resin. This observation was common to all specimens evaluated. Evaluating a larger number of samples is crucial to confirm this finding, as is the association of tests directly related to this issue, such as water sorption tests, hydrolytic degradation tests, and mechanical resistance tests of this material.

This work presents intrinsic limitations of *in vitro* tests. Therefore, the data obtained helped to understand the behavior of the adhesive systems studied without providing all the variables present clinically.

Based on the results of the present study, the chemically activated adhesive (Stela Primer/SDI) exhibited similar shear bond strengths to those of the universal self-etching adhesive with 10-MDP (Single Bond Universal/3M). The previous application of air blast particle abrasion (APA) significantly increased shear bond strength in both adhesive systems. Finally, the increase in bond strength provided by the previous APA application was similar for both adhesive systems.
